# Supplementary material for: Optimization based data enrichment using stochastic dynamical system models
Source: PLoS One. 2024 Sep 20;19(9):e0310504. doi: 10.1371/journal.pone.0310504 (PMC11414895; doi:10.1371/journal.pone.0310504)
Supplement: S1 Appendix — We include proof of the extension theorem which is used to generate stochastic distributions on continuous time intervals. We review the application of the calculus of variations which is used to derive the requirements included in the main theorem of the present work. (PDF) [file pone.0310504.s001.pdf]

Extension of Distributions

In discussion of the preliminaries we introduced an infinite product as a route toward defining the distribution extension, but this approach is intractable. Instead, we prescribe mathematically meaningful properties for μ, and prove that these induce the expression (4).

We require three properties: Constancy, Monotonicity, and Geometric Averaging:

Definition 1 *(Constancy) If $\rho = c$ is constant on the interval τ, then $\mu(\tau, \rho) = c$.*

Definition 2 *(Monotonicity) If $\rho \geq \hat{\rho}$ on the interval τ, then $\mu(\tau, \rho) \geq \mu(\tau, \hat{\rho})$.*

Definition 3 *(Geometric Averaging) If τ_1 and τ_2 are a partition of the continuous interval τ such that $\tau = \tau_1 \cup \tau_2$, then*

$$\mu(\tau, \rho)^{|\tau|} = \mu(\tau_1, \rho)^{|\tau_1|} \mu(\tau_2, \rho)^{|\tau_2|}.$$

We show that these properties induce the extension expression used in this work,

Theorem 1 *Assume $\ln \rho$ is Lebesgue integrable on τ. $\mu(\tau, \rho)$ satisfies constancy, monotonicity, and geometric averaging if and only if*

$$\mu(\tau, \rho) = e^{\frac{1}{|\tau|} \int_{\tau} \ln \rho \ dt},$$

and we call Theorem 1 the *extension theorem*.

The proof of the theorem uses concepts from Lebesgue measure and Lebesgue integration. We do not cover these notions and instead refer to [12] for details.

Proof 1 ***Forward Direction:***
Assume that $\mu(\tau, \rho)$ satisfies constancy, monotonicity, and geometric averaging. Let

$$s = \sum_{n=0}^{N-1} s_n 1_{\tau_n}$$

be a simple function on τ, where $\{s_n\}_{n=0}^{N-1}$ is a set of real scalars and

$$1_{\tau_n} = \begin{cases} 1 & t \in \tau_n \\ 0 & t \notin \tau_n. \end{cases}$$

The set $\{\tau_n\}_{n=0}^{N-1}$ is a covering of τ. We use the geometric averaging and constancy properties to compute $\mu(\tau, s)$ by writing

$$\mu(\tau, s)^{|\tau|} = \prod_{n=0}^{N-1} s_n^{|\tau_n|},$$

noting that $\mu(\tau_n, s) = s_n$ by construction. Taking the logarithm of this expression yields

$$|\tau| \ln \mu(\tau, s) = \sum_{n=0}^{N-1} |\tau_n| \ln(s_n)$$

in general for simple functions. The expression on the right hand side of this equation is aligned with the Lebesgue definition of the integral of the logarithm of the simple function,

$$\sum_{n=0}^{N-1} |\tau_n| \ln(s_n) = \int_\tau \ln s \, dt.$$

The strategy will be to approximate $\ln\rho$ *above and below by simple functions which can be used to approximate the integral in the Lebesgue sense to arbitrary precision. Given* $\epsilon > 0$*, since* $\ln\rho$ *is measurable, there is a simple function* $s^* \geq \rho$ *such that*

$$|\tau|\ln\mu(\tau,\rho) \leq |\tau|\ln\mu(\tau,s^*) = \int_\tau \ln s^* dt < \int_\tau \ln\rho \, dt + \epsilon.$$

Similarly, there is a simple function $s_* \leq \rho$ *such that*

$$\int_\tau \ln\rho \, dt - \epsilon < \int_\tau \ln s_* dt = |\tau|\ln\mu(\tau,s_*) \leq |\tau|\ln\mu(\tau,\rho).$$

Therefore,

$$\left| |\tau|\ln\mu(\tau,\rho) - \int_\tau \ln\rho \, dt \right| < \epsilon$$

and we conclude that

$$\ln\mu(\tau,\rho) = \frac{1}{|\tau|}\int_\tau \ln\rho \, dt,$$

as ϵ *is arbitrary.*

Reverse Direction:

Assume

$$\mu(\tau,\rho) = e^{\frac{1}{|\tau|}\int_\tau \ln\rho \, dt}.$$

We show the properties hold directly. For constancy, assume that $\rho = c$ *on* τ *and consider*

$$\begin{aligned}\mu(\tau,\rho) &= e^{\frac{1}{|\tau|}\int_\tau \ln\rho \, dt} \\ &= e^{\frac{1}{|\tau|}\int_\tau \ln c \, dt} \\ &= e^{\frac{\ln c}{|\tau|}\int_\tau dt} \\ &= e^{\ln c} \\ &= c.\end{aligned}$$

Monotonicity follows from properties of the integral, logarithm, and exponential:

$$\frac{1}{|\tau|}\int_\tau \ln\rho dt \geq \frac{1}{|\tau|}\int_\tau \ln\hat{\rho} dt$$

for all $\rho \geq \hat{\rho}$*. Therefore,*

$$e^{\frac{1}{|\tau|}\int_\tau \ln\rho dt} \geq e^{\frac{1}{|\tau|}\int_\tau \ln\hat{\rho} dt}.$$

Finally, we show geometric averaging holds by taking $\tau = \tau_1 \cup \tau_2$ *for some* τ_1 *and* τ_2*, and considering*

$$\begin{aligned}\mu(\tau,\rho)^{|\tau|} &= e^{\int_\tau \ln\rho \, dt} \\ &= e^{\int_{\tau_1} \ln\rho \, dt + \int_{\tau_2} \ln\rho \, dt} \\ &= e^{\int_{\tau_1} \ln\rho \, dt} e^{\int_{\tau_2} \ln\rho \, dt} \\ &= \mu(\tau_1,\rho)^{|\tau_1|}\mu(\tau_2,\rho)^{|\tau_2|}.\end{aligned}$$

This completes the proof.

Application of the Calculus of Variations

We provide a summary of the analysis of problem (22) through demonstration of the calculus of variations. For each of the variables in the optimization we introduce a variation. For instance for x,

$$\tilde{x} = x + \delta x,$$

where $\tilde{x}$ is the perturbed state, x is the unperturbed state, and δx is the variation.

We describe the procedure for a simple, but representative, objective that is written as

$$J(u) = \sum_{k=0}^{K-1} \int_{t_k}^{t_{k+1}} F(u, \dot{u}, t)dt + \sum_{k=0}^{K} G(u(t_k), \dot{u}(t_k), t_k).$$

For the purposes of the summary we use u as a general variable, and in (22) the general variable is constructed by taking

$$u = \begin{pmatrix} x \\ v \\ w \\ \lambda \\ \eta \end{pmatrix}.$$

Using standard techniques in the CoV allows us to compute the variation of the integral term

$$\int_{t_k}^{t_{k+1}} F dt$$

via

$$F(u + \delta u, \dot{u} + \delta \dot{u}, t) = F(u, \dot{u}) + \delta F(u, \dot{u}) + \text{h.o.t},$$

where

$$\delta F(u, \dot{u}) = \frac{\partial F}{\partial u}(u, \dot{u})\delta u + \frac{\partial F}{\partial \dot{u}}(u, \dot{u})\delta \dot{u},$$

to obtain

$$\frac{\partial F}{\partial \dot{u}}\delta u \mid_{t_{k+1}^-} - \frac{\partial F}{\partial \dot{u}}\delta u \mid_{t_k^+} - \int_{t_k}^{t_{k+1}} (\frac{d}{dt}\frac{\partial F}{\partial \dot{u}} - \frac{\partial F}{\partial u})\delta u \, dt.$$

In this expression we use the notation

$$\frac{\partial F}{\partial \dot{u}}\delta u \mid_{t_k^+} = \lim_{t \to t_k^+} \frac{\partial F}{\partial \dot{u}}(t)\delta u(t)$$

and employ integration-by-parts to remove all temporal derivatives from the variation δu.

The variation of the discrete term

$$G(u(t_k), \dot{u}(t_k), t_k)$$

is written as

$$\frac{\partial G}{\partial u}\delta u \mid_{t_k} + \frac{\partial G}{\partial \dot{u}}\delta \dot{u} \mid_{t_k}.$$

Taking the sum of these variations (over k) and collecting similar terms yields the conditions

$$\frac{d}{dt}\frac{\partial F}{\partial \dot{u}}(t) - \frac{\partial F}{\partial u}(t) = 0 \tag{1}$$

for all $t \in \mathscr{T} - \mathscr{T}_M$,

$$-\frac{\partial F}{\partial \dot{u}}(t_k^+) + \frac{\partial F}{\partial \dot{u}}(t_k^-) + \frac{\partial G}{\partial u}(t_k) = 0, \tag{2}$$

and

$$\frac{\partial G}{\partial \dot{u}}(t_k) = 0 \tag{3}$$

for all $t_k \in \mathscr{T}_M$. In these expressions we have enforced that the variations must vanish for all $\delta u \neq 0$, for otherwise the objective would not be at a critical point and the solution would not be optimal. Equation (1), when applied to the first term on the right of (21), generates (23)–(25) in Theorem 1, and Equations (2) and (3), when applied to the second term on the right of (21), generate (26)–(31).
