# Supplementary material for: Optimization based data enrichment using stochastic dynamical system models
Source: PLoS One. 2024 Sep 20;19(9):e0310504. doi: 10.1371/journal.pone.0310504 (PMC11414895; doi:10.1371/journal.pone.0310504)
Supplement: Attachment — Submitted filename: Response to Reviewers.pdf [file pone.0310504.s002.pdf]

OpB Data Insights, LLC
168 Ridgeway Avenue • Syracuse, NY, 13224 • USA

Dear Editor,

Please, find enclosed our revised manuscript entitled "Optimization Based Data Enrichment Using Stochastic Dynamical System Models" which we are gratefully resubmitting for publication in PLoS One per your invitation to do so following an initial peer review cycle. We found the reviewers' comments helpful, insightful, beneficial, and we have done our best to address each of the points that were raised as follows.

Reviewer 1:

1. Reviewer comment: The literature review must be expanded.
   Revision action: You will see substantial additions to and expansion of the introduction of the manuscript, including added contextual discussion on another related method, Gaussian Process Regression, with additional literature references.

2. Reviewer comment: Readers may not be well-versed in cubic splines.
   Revision action: You will find additional description of cubic splines in the introduction along with an additional reference (Donohue 2023) that covers an application of a specific type of existing cubic spline we discuss as a related method. We also added additional review of the definition of a cubic spline in the body of the work on page 8 before previewing where they naturally arise in our newly proposed method.

Reviewer 2:

1. Reviewer comment: Data should be added to the abstract to show effectiveness.
   Revision action: We found it difficult to efficiently incorporate quantitative data discussion in the abstract itself without making its length unreasonable due to that nature of the work. In lieu of this, and in an effort to add language about data inclusion to the abstract in some capacity, we indicate the inclusion of a Monte Carlo simulation in the body of the work which demonstrates that our method realizes significant improvement in comparison to another existing method. It was due to the suggestions of this reviewer that we designed the Monte Carlo simulation and included it to explicitly quantify the performance of our technique. The Monte Carlo simulation and the accompanying analysis is found on pages 13 through 15 in the revised manuscript.

2. Reviewer comment: The drawbacks of the conventional techniques should be discussed in the introduction.
   Revision action: In the expanded introduction we now call out the drawbacks of existing techniques explicitly.

3. Reviewer comment: Reference all equations.
   Revision action: All equation are now endowed with a reference per the reviewer request.

4. Reviewer comment: In the conclusion add an enhancement percentage for the proposed algorithm when used with another method.
   Revision action: Using the Monte Carlo simulation discussed above we are able to quantitatively compare our proposed method to the alternative existing method of natural cubic splines. We include the enhancement percentage observed using the simulated data.

Reviewer 3:

1. Reviewer comment: Inclusion of a graphical abstract.
   Author response: We would be happy to include a graphical abstract to add clarity during publication edits, assuming the manuscript is accepted.

We hope that you find these revisions satisfactory. We reiterate that we are grateful for the review feedback as we believe the suggested changes have strengthened the quality of the manuscript. We look forward to your next correspondence.

Thank you,
Griffin M. Kearney
